# Ulcerative colitis: understanding its cellular pathology could provide insights into novel therapies

**DOI:** 10.1186/s12950-020-00246-4

**Published:** 2020-04-21

**Authors:** Amandip Kaur, Paraskevi Goggolidou

**Affiliations:** Department of Biomedical Science and Physiology, Faculty of Science and Engineering, University of Wolverhampton, Wulfruna Street, Wolverhampton, WV1 1LY UK

**Keywords:** Ulcerative colitis, Cytotoxic T-lymphocyte antigen 4 (CTLA4), IL-10, Tumour necrosis factor alpha (TNF-α), Faecal microbiota transplantation

## Abstract

Dynamic interactions between the gastrointestinal epithelium and the mucosal immune system normally contribute to ensuring intestinal homeostasis and optimal immunosurveillance, but destabilisation of these interactions in genetically predisposed individuals can lead to the development of chronic inflammatory diseases. Ulcerative colitis is one of the main types of inflammatory diseases that affect the bowel, but its pathogenesis has yet to be completely defined. Several genetic factors and other inflammation-related genes are implicated in mediating the inflammation and development of the disease. Some susceptibility loci associated with increased risk of ulcerative colitis are found to be implicated in mucosal barrier function. Different biomarkers that cause damage to the colonic mucosa can be detected in patients, including perinuclear ANCA, which is also useful in distinguishing ulcerative colitis from other colitides. The choice of treatment for ulcerative colitis depends on disease severity. Therapeutic strategies include anti-tumour necrosis factor alpha (TNF-α) monoclonal antibodies used to block the production of TNF-α that mediates intestinal tract inflammation, an anti-adhesion drug that prevents lymphocyte infiltration from the blood into the inflamed gut, inhibitors of JAK1 and JAK3 that suppress the innate immune cell signalling and interferons α/β which stimulate the production of anti-inflammatory cytokines, as well as faecal microbiota transplantation. Although further research is still required to fully dissect the pathophysiology of ulcerative colitis, understanding its cellular pathology and molecular mechanisms has already proven beneficial and it has got the potential to identify further novel, effective targets for therapy and reduce the burden of this chronic disease.

The gastrointestinal (GI) tract, found in humans and animals, represents a unique environment extending from the mouth to the anus [1]. The intestine, a muscular tube of the digestive system stretching from the stomach to the anus, consists of the small and large intestine. It is involved in food digestion as well as in enzyme and hormone production (e.g. cholecystokinin that stimulates the secretion of pancreatic enzymes and bile). It plays an important role in fighting pathogens and in regulating the body’s water balance [2] and it has been demonstrated that the gut microflora potentially contributes to proteolysis in the human colon [3]. The large intestine, which is involved in the transport of water and electrolytes and the storage of faecal waste in the sigmoid colon and rectum prior to elimination [1], is implicated in the processing of indigestible food after most nutrients are absorbed in the small intestine [4].

Many disorders affect the colon’s ability to work properly. Inflammatory Bowel Disease (IBD) is a term used to describe disorders that involve chronic inflammation of the digestive tract, which include both ulcerative colitis (UC) and Crohn’s disease (CD), another chronic inflammatory disease that causes inflammation of the full thickness of the bowel wall [5, 6]. Although strides are being made in better understanding IBD, it is important to shed light on its individual manifestations and for this purpose, this article will focus on UC.

## Ulcerative colitis and large intestine physiology

UC is a disease of unknown aetiology characterized by inflammation of the mucosa and sub-mucosa of the colon and rectum lining, causing ulcers to develop. It is usually possible to notice a clear margin between normal and affected intestinal tissue [7]. There has been a global increase in UC incidence, with the highest incidence in the West observed in Canada (16.7 per 100,000 people) [8], while in Europe UC incidence ranges from 1.6 to 11.9 per 100,000 people, with more patients observed in Northern European countries [9]. In the East, although UC incidence is increasing, it is rarer with the greatest incidence observed in Korea (3.62 per 100,000) [10]. Globally, due to its chronic nature and the low mortality observed in UC, its prevalence has increased and can reach up to 294 per 100,000 in Europe [11]. Certain ethnic groups are more prone to UC, but environmental factors, such as smoking, oral contraceptives, diet, antibiotics, vaccination, infections and childhood hygiene also play a role [12]. UC may affect any age group, with the peak age of diagnosis ranging from 15 to 40 years of age and with most UC studies showing equal gender distribution [13]. UC often presents with blood in the stool and diarrhoea. Common symptoms include urgency, incontinence, fatigue, increased frequency of bowel movements, mucus discharge, nocturnal defecations and abdominal discomfort; fever and weight loss can also be noticed. These clinical presentations may vary depending on disease severity.

**Fig. 1:**
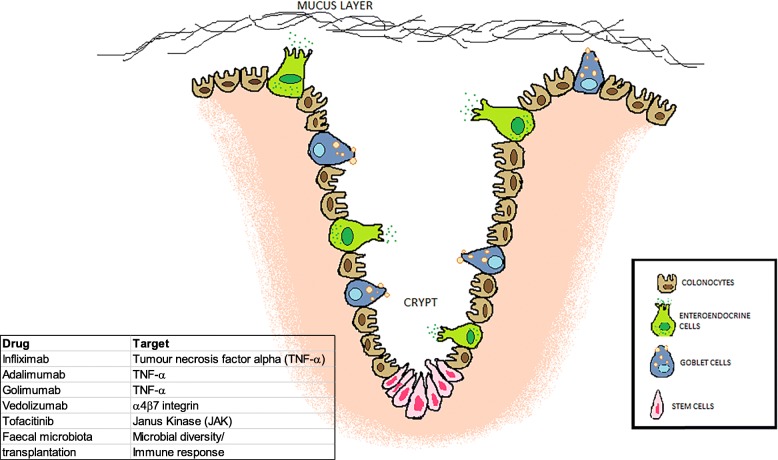
The cross-sectional crypt structure of the large intestine and current UC therapeutic strategies. The intestinal epithelium is lined with a single layer of polarized cells, whose major cell types include colonocytes, enteroendocrine, goblet cells and stem cells

To dissect the molecular basis of UC, it is necessary to have thorough knowledge of the cellular populations that constitute the large intestine. The mucosa of the colon is lined by a single-layered columnar epithelium with a thin brush border that is essential for maintaining gut homeostasis and functions as a physical and biochemical barrier and a coordinating centre for immune defense and crosstalk between bacteria and immune cells. It consists of invaginations known as ‘crypts of Lieberkühn’ (Fig. 1). Intestinal stem cells, which are responsible for the rapid renewal of the intestinal epithelium, reside at the base of these crypts and develop into transient proliferative cells that differentiate as they travel through the transition zone, where intestinal epithelial cells eventually shed into the lumen at the apex of crypts [14]. Intestinal epithelial stem cells can specialize into many cell types including enterocytes, Paneth cells, goblet cells, and neuroendocrine cells [15]. Most of the cells present in the intestine are absorptive cells, with the exception of crypt cells that are principally secretory cells [2].

Colonocytes are the most abundant cell type in the large intestine and they are involved in electrolyte absorption though passive diffusion of lipid-soluble molecules [16]. Goblet and enteroendocrine cells are secretive cells. Goblet cells are specialized epithelial cells, which are found in the non-follicle bearing epithelium of the intestine and they comprise around 10% of all intestinal epithelial cells. They have an important role in innate immunity by synthesizing and releasing mucin, a viscous fluid enriched in mucin glycoproteins that form large net-like polymers that lubricate the lumen to promote movement and effective diffusion of gut contents. Goblet cells also act as a physical barrier, protecting the intestinal wall from digestive enzymes and bacterial adhesion to the underlining epithelium. Although goblet cells are present in both the small and large intestine, they are more abundant in the large intestine due to the greater numbers of intestinal bacteria [17]. Goblet cells also produce and secrete biologically active substances that contribute to innate immunity, such as trefoil peptides, resistin-like molecule β (RELMβ) and Fc-γ binding protein (Fcgbp), which respectively function to promote epithelial restitution, inhibit intestinal nematode chemotaxis and stabilize the mucous layer [18].

Enteroendocrine cells, which produce and secrete hormones, consist of 1% of the large intestinal epithelium [19]. One of these molecules is vasoactive intestinal peptide (VIP), a peptide hormone that inhibits gastrin release and acid secretion and stimulates water and electrolyte secretion by the small and large intestines. Because VIP plays an essential role in regulating colonic mucosal integrity and epithelial barrier homeostasis, alterations in VIP tissue concentration are associated with increased colitis susceptibility [20]. Correct functioning of specialized intestinal epithelial cells is essential to maintain intestinal homeostasis and its dysfunction plays a central role in the pathogenesis of several diseases, including UC [21]. A common histological pattern identified in UC is the architectural distortion of the intestinal epithelium characterised by the shortening and reduced branching of the crypts [22]. This microscopic change in chronic UC can be detected in every biopsy fragment from the diseased colon [23]. The lamina propria of the large intestine also contains immune cells, including macrophages, dendritic cells, plasma cells, and lamina propria lymphocytes [24]. These immune cells together with UC patients’ genetic predisposition play a crucial role in UC progression.

## Genetics and immunological response involvement in UC

UC is a multifactorial disorder; genetic predisposition, epithelial barrier defects, dysregulated immune responses and environmental factors play a role in its pathogenesis. A recent meta-analysis of genome-wide association studies (GWAS) has identified 163 IBD-associated loci linked to both UC and CD. These loci contain genes involved in autophagy, microbe recognition, lymphocyte signalling, response to endoplasmic reticulum stress and cytokine signalling [25]. Although the exact aetiology of UC remains elusive, the commensal luminal flora is known to trigger an inappropriate and overactive mucosal immune response in genetically susceptible individuals, causing intestinal tissue damage.

The meta-analysis of GWAS has found many novel single nucleotide polymorphisms (SNPs) for UC, which is believed to be more genetically heterogeneous than CD. 163 risk loci have been identified, of which 110 confer common susceptibility to IBD; hence, these SNPs are associated with both disease phenotypes, whereas 30 seem to be specific to CD and 23 to UC [26]. These UC SNPS are found in genes implicated in mucosal barrier function, such as *Extracellular Matrix 1 (ECM1), Cadherin Type 1 (CDH1), Hepatocyte Nuclear Factor 4 alpha (HNF4α) and Laminin Beta 1* (*LAMB1*; Table 1). Polymorphisms in *Interleukin 10 (IL-10)* correlate with impaired IL-10 production that confers UC risk [27]. The majority of molecular differences between UC and CD are found in human leukocyte antigen (HLA) Class II genes and in genes associated with binding pattern recognition [30]. These include nucleotide-binding oligomerization domains (NODs) and toll-like receptors (TLRs), innate immunity, (IL-23R) and autophagy pathways (ATG16L1, IRGM). HLA class II genes *DR2*, *DR9*, and DRB1*0103, were shown to be UC susceptibility genes, in fact, DRB1*0103 is significantly associated with disease susceptibility, extensive disease and an increased risk of colectomy [30]. On the other hand, the HLA class II gene *DR4* was a protective gene in UC [30].

**Table 1:** The genes implicated in mucosal barrier function that confer risk to UC [27–29]

GENE	LOCUS	SNP	PROTEIN NAME	FUNCITON
*ECM1*	1q21	rs3737240	Extracellular matrix protein 1	Glycoprotein involved in cell proliferation
*CDH1*	16q22	rs12597188	E-cadherin	Protein involved in epithelial adherens junction
*HNF4A*	20q13	rs6017342	Hepatocyte nuclear factor 4α	Transcriptional factor that regulates cellular differentiation along crypt-villus axis
*LAMB1*	7q31	rs886774	Laminin β1	Protein involved in cell adhesion and differentiation
*IL10*	1q32	rs3024505	Interleukin 10	Anti-inflammatory cytokine

In addition, Cytotoxic T-lymphocyte antigen 4 (CTLA4) is an inhibitory receptor expressed by activated T cells and an important downregulator of T cell activation, as it suppresses T cell effector function following initial activation by co-stimulatory signals [31]. CTLA4 plays a critical role in the priming phase of the immune response and it might also contribute to peripheral tolerance. Because *CTLA4* has an important role as a negative regulator of T cell activation and monocyte-macrophage cognate interaction, it is considered a good candidate gene for UC susceptibility. Several genetic polymorphisms have been reported in the human *CTLA4* gene [30]. One such study was performed on 87 Chinese UC patients that were genotyped for *CTLA-4 promoter − 1661* and *A-1661G* non-exonic region polymorphisms. It was concluded that the *A-1161G CTLA4* polymorphism is a UC risk factor in Chinese patients [31].

Besides the genetic profile of UC patients, it is important to note that the disease itself involves dysregulated immune responses against intraluminal and mucosal antigens, which usually include commensal bacteria [32]. It is believed that the chronic inflammatory response arises following a pathogenic organism infection such as *Shigella spp.* or *Campylobacter spp*., which remains in the intestinal tissues [33]. Exposure to microbial peptides that share immunogenic elements with self-antigens induces immune tolerance disruption to endogenous gut antigens. Thus, a possible underlying basis for UC is a destructive inflammatory response directed towards self-antigens such as mucin, goblet cells and colonocytes [34].

Autoantibodies in the mucosa of the large intestine may play a part in the pathogenesis of this disease. The local production of these autoantibodies is stimulated by T-cell abnormalities that reside within the epithelial cell layer and the lamina propria of the large intestine and the associated activation of antibody-producing cells [35]. The autoantibodies detected in the serum of UC patients include the anti-colon antibody and the anti-neutrophil cytoplasmic antibody (ANCA) [35]. These are involved in antibody-dependent cell-mediated cytotoxicity (ADCC), which is presumed to be the cause of damage that occurs to colonic mucosa [36]. Levels of disease-specific autoantibodies to a neutrophil protein with a perinuclear distribution, pANCA, reflect the extent of the immune response associated with UC. However, these antibodies may develop following an infection; hence, there is not enough evidence to support the correlation between these autoantibodies and the pathogenesis of the disease [36].

One recurrent UC feature is neutrophil accumulation in the inflamed intestinal mucosa. Neutrophil granulocytes contain enzymes, one of which is myeloperoxidase (MPO). This granule enzyme is released upon stimulation with cytotoxic oxygen metabolites. Therefore, activated neutrophils may contribute to tissue damage at sites of inflammation. It has been shown that MPO concentrations were increased several fold in UC patients compared to healthy controls, which is indicative of enhanced neutrophil activity [36]. Faecal MPO assessment is a simple, non-invasive marker of disease and inflammation activity. Low stool MPO levels can detect intestinal healing and it is an early marker of treatment response in UC patients, while high levels can predict relapse [36].

There is also considerable evidence that defective mucosal immunoregulation, including abnormal changes of T cells, B cells, granulocytes, macrophages and the cytokines and chemokines produced by these cells, plays a major role in UC pathogenesis [35]. One of the consistently replicated markers found in UC patients is the SNP rs3024505, which immediately flanks *IL10* on chromosome 1q32.1 [37]. Polymorphisms in *IL-10* are associated with loss-of-function mutations in *IL-10* and *IL-10 receptor* and are characteristic of early UC onset [38]. IL10 is an immunosuppressive cytokine produced by B cells, T cells, macrophages and some non-haematopoietic cells upon stimulation [39]. IL-10 has a broad effect in immunoregulation and host defense, as it affects both the innate and adaptive immune systems [40]. Macrophage-derived IL-10 was shown to be dispensable for mouse gut homeostasis, while IL-10 receptor deletion resulted in the manifestation of severe colitis due to monocyte-derived macrophages impairement [41]. Pro-inflammatory cytokines that should be suppressed by IL-10 can be regulated by nuclear factor kappa-light-chain-enhancer of activated B cells (NF-κB). Abnormal activation of NF-κB and impaired production of IL-10 have been proposed to influence UC pathophysiology [42].

## The role of biomarkers and treatment options in UC

The variable immunological responses and complex genetics of UC pose a significant problem to the clinical and scientific community, with regards to identifying a suitable treatment strategy for all patients. A number of approaches have been attempted in the past decade and various clinical trials are underway, in order to identify treatments that will allow all patients to quickly reach and remain in remission after periods of flare-ups. A uniform approach for all UC patients is however proving quite challenging and as such, a tendency towards personalised treatment and care approaches is rapidly gaining ground. Assisting towards this goal, the identification of specific biomarkers could help predict UC’s course and identify specific pathways involved in disease progression and improved treatment [43, 44]. A known UC serum diagnostic biomarker is pANCA, found in 50–75% of UC patients. pANCA staining distinguishes UC from CD and other colitides and provides a prognostic feature of the risk of developing refractory pouchitis after colectomy [45]. However, pANCA can also identify an antigen expressed by bacteria resident in the human colonic mucosa, therefore some bacterial proteins cross-react to pANCA epitopes. It was observed that UC patients with high pANCA titers, had higher anti-OmpC *E.coli* IgG levels than healthy controls [36]. The cross-reactivity of serum UC pANCA with *E. coli* membrane protein OmpC, suggests that enteric bacterial proteins are involved in UC pathogenesis [33]. It should be noted that 32% of healthy controls were tested positive for pANCA, limiting the diagnostic value of this biomarker [44]. In addition to biomarkers, an exponential increase in the number of novel therapeutic UC targets has been observed in the past decade, although it is interesting to note that their efficiency varies in the UC patient population, highlighting the need for personalised medicine interventions. Infliximab, adalimumab and golimumab are the anti-tumour necrosis factor alpha (TNF-α) monoclonal antibodies available in the UK for the treatment of UC in adults, but they can also be used to treat other immune-mediated disorders such as rheumatoid arthritis, ankylosing spondylitis, psoriasis, hidradenitis suppurativa and refractory asthma [46]. TNF-α is an inflammatory cytokine produced by macrophages and monocytes during acute inflammation and it is involved in inflammation, apoptosis, stimulation of lymphocytes and activation of immune cell functions [47]. TNF-α is one of the most important cytokines that mediates intestinal tract inflammation and increased TNF-α expression is detected in UC patients [48]. Clinical trials showed that treatment with TNF-α inhibitors results in a significantly higher rate of clinical response, clinical remission and mucosal healing in UC [48], nevertheless, although TNF-α inhibitors are effective in a proportion of UC patients, their mechanisms of action in UC remain largely unknown. Increased mononuclear phagocyte populations were observed in non-responder UC patients pre and post-infliximab treatment [49], potentially shedding some light into why UC patients, who initially respond to infliximab treatment, lose response or become resistant over time. Although data on the adverse effects of infliximab in UC patients is limited, infectious complications such as bacterial pneumonia, tuberculosis and opportunistic infections can occur during therapy [48].

The adhesion of T lymphocytes from the peripheral circulation to the gut mucosa is a central step for the progression of the inflammatory process in UC [50]. Different anti-adhesion agents have been suggested for UC treatment. Vedolizumab is a humanised monoclonal IgG-1 antibody that selectively inhibits α4β7 integrin and mucosal addressin cell adhesion molecule-1 (MAdCAM-1) interaction. It prevents lymphocyte infiltration from the blood into the inflamed gut tissue, reducing local inflammation [51]. In addition to this effect, vedolizumab also reduces α4β7-dependent gut homing of non-classical monocytes, resulting in a decrease in alternatively activated M2-like macrophages in the gut [52]. In contrast to other anti-adhesion drugs, the use of vedolizumab in UC patients did not increase the rates of opportunistic or enteric infections and there were no reported cases of progressive multifocal leukoencephalopathy [53]. Following encouraging results in randomized, double-blind, placebo-controlled trials in the pivotal phase III GEMINI studies, vedolizumab has been approved by US FDA for the treatment of adult patients with active UC who had a poor response to standard therapies [54]. Nevertheless, mononuclear phagocyte enrichment was detected in non-responder UC patients before vedolizumab treatment, which further increased post treatment [52], partly explaining why some UC patients do not respond as well to this drug.

Another important therapeutic target is the Janus kinase (JAK) family of tyrosine kinases, which contains four members JAK1, JAK2, JAK3 and TYK2 that are responsible for mediating signal transduction for many cytokine receptors including interleukins (ILs) 2, 4, 6, 7, 9, 12, 15 and 21 [55]. Tofacitinib is a novel selective inhibitor of JAK1 and JAK3 and, to a lesser extent, JAK2 [56],with Phase 3 trials showing a significant amelioration in symptoms in moderate and severe UC patients [57]. This oral drug works by suppressing the differentiation of pathogenic Th1 and Th17 cells and innate immune cell signalling and it was demonstrated to be efficient in inducing and maintaining remission and achieving mucosal healing in patients with moderately to severely active UC [57].

Different pilot clinical trials have evaluated the efficacy of type I IFN-α and IFN-β (IFN-α/β) in active UC, delivering promising results. IFN-α/β is involved in stimulating the production of anti-inflammatory cytokine IL-10 by CD4+ T cells. IFN-α/β also plays a role in the modulation of Th1 responses and it inhibits production of Th2 cytokines, IL-5 and IL-13 that are upregulated in the mucosa of UC patients [58]. However, the majority of patients treated with IFN-α/β experienced adverse events such as headache, arthralgia, myalgia, abdominal pain, fatigue and vomiting [59].

Another therapeutic intervention involves phospholipids, the components of the GI mucus which they are indispensable for intact barrier function [60]. Phosphatidylcholine (PC) is the major mucus phospholipid and was significantly reduced in the mucus of UC patients compared to healthy controls [61]. Lack of PC could enable the invasion of luminal noxious agents into the gut mucosa [61]. Hence, there is the hypothesis that PC reconstitution in the colonic mucus of UC patients could help to re-establish the structure and density of the mucus, enhance mucus barrier function and prevent ulterior inflammation in UC. Oral daily administration of a PC-rich phospholipid preparation could be an innovative therapeutic approach that helps with remission in moderate UC patients, without the significant side effects that are usually seen with the usage of steroid or immunosuppressive therapy [55].

Alternative therapies, such as probiotics (*Escherichia coli* Nissle), can also be considered in preventing UC relapse. Probiotics act as a barrier, as they line the intestinal tract and through competitive inhibition, prevent other luminal bacteria from reaching the lamina propria and stimulating the mucosal immune system [62]. Probiotics also enhance mucus production which protects against invasive bacteria, induce protective cytokines and suppress pro-inflammatory cytokines and can modulate the immune system in the gut [63, 64]. In addition, faecal microbiota transplantation (FMT), the transfer of stool from a healthy donor to a UC patient is emerging as a promising approach to alleviating UC severity. FMT has been shown to result in increased secretory IgA and mucin as well as anti-microbial peptide production, affecting pathogen invasion by antigen/pathogen-dependent and –independent targeting [65]. In the case of UC, a few randomised controlled trials are currently underway, with one recent study showing that some UC patients could achieve remission following continuous FMT thanks to the observed greater microbial diversity and enrichment of *Eubacterium hallii* and *Roseburia inulivorans* in faecal and colon samples [66]. Significantly, the immunological outcomes of FMT are hard to dissect. A randomised clinical trial failed to identify any significant changes in γδ T cells, natural killer cell or overall T cell ratios, although they discovered a slight increase in gut-homing CD4 cells [67]. A study of moderate to severe UC patients did not identify any changes in serum cytokines (including IL-10 and IL-17) post single upper GI FMT delivery [68], while another single FMT study detected a reduction in colonic mucosal Th1 and Treg cells post-FMT, but no difference in the Th17 cell population [69]. It thus transpires that repeated FMT might be necessary for successful UC remission and the choice of donors with the appropriate microbiome might prove beneficial in improving UC patient outcomes.

## Conclusions

It becomes obvious from the above that the identification of specific biomarkers and increased knowledge of the immunological and cellular mechanisms of the disease can contribute to better understanding of UC pathogenesis. Elevated mononuclear phagocyte populations in UC colonic mucosa could partly explain why some UC patients respond well to infliximab and vedolizumab treatment and others do not. Although the above-mentioned drugs and treatment strategies have been shown to be fairly effective, the next challenge would be to develop targeted and personalised therapies for UC patients, potentially also taking advantage of the genetic and cellular technology advancements in IBD. It remains to be seen whether these approaches will be effective for the wide spectrum of UC patients, but the recent advances in personalised medicine create endless opportunities for the future of UC diagnosis and prognosis.

## Data Availability

Not applicable.

## Abbreviations

ANCA: Anti-neutrophil cytoplasmic antibody
CD: Crohn’s disease
GWAS: Genome-wide association studies
HLA: Human leukocyte antigen
IBD: Inflammatory bowel disease
IFN: Interferon
IL: Interleukin
JAK: Janus kinase
MHC: Major histocompatibility complex
MPO: Myeloperoxidase
NF-κB: Nuclear factor kappa-light-chain-enhancer of activated B cells
PC: Phosphatidylcholine
pANCA: Perinuclear anti-neutrophil cytoplasmic antibody
SNP: Single nucleotide polymorphism
TNF: Tumour necrosis factor alpha
TNFα: Anti-tumour necrosis factor alpha
UC: Ulcerative colitis
VIP: Vasoactive intestinal peptide
